# A Case of Human Viral Encephalitis Caused by Pseudorabies Virus Infection in China

**DOI:** 10.3389/fneur.2019.00534

**Published:** 2019-06-04

**Authors:** HongNa Yang, Hui Han, Hao Wang, Yi Cui, Han Liu, ShiFang Ding

**Affiliations:** Department of Critical-Care Medicine, Qilu Hospital of Shandong University, Shandong University, Jinan, China

**Keywords:** pseudorabies virus, viral encephalitis, herpes simplex encephalitis, Japanese encephalitis, next-generation sequencing

## Abstract

We report a human case of viral encephalitis caused by pseudorabies virus (PRV) in China. A 43-year-old man with no previous medical history presented with high-grade fever, headache and tonic-clonic seizures as well as coma. Plain computer tomography (CT) brain imaging showed hypo-density in the bilateral basal ganglia, bilateral occipital lobe, bilateral limbic lobe, and left thalamic. Next-generation sequencing (NGS) confirmed the presence of PRV in cerebral spinal fluid (CSF). Regular polymerase chain reaction (PCR) was applied to confirm the presence of PRV in the CSF and blood. In addition, serological (immunological) tests were used to further validate the presence of PRV in the peripheral blood. This case suggested that it was possible for PRV to result in human central nervous system (CNS) infection, and it is necessary for people to increase awareness of self-protection when contacting animals.

## Background

Pseudorabies virus (PRV; also called Aujeszky's disease or Suid herpesvirus type 1), which belongs to the Alphaherpesvirinae subfamily and genus Varicellovirus in the family Herpesviridae, causes fatal encephalitis in newborn piglets, respiratory disorders in growing-fattening pigs, and reproductive failure in sows. In the past, PRV was thought to not infect humans and just infect mammals including pigs, cattle, sheep, goats, dogs, cats, foxes, rats, and wild mice (1). Recently, Ai et al. reported a case of human endophthalmitis caused by PRV infection (2), which suggested that it was possible for PRV to infect humans. Here, we reported a case of encephalitis caused by PRV.

## Case Presentation

**Figure 1:**
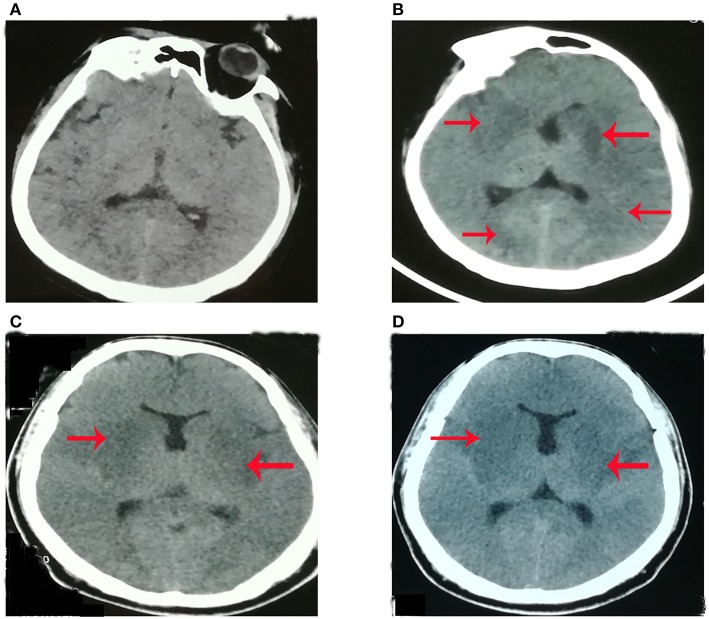
The plain brain CT imaging at different time [**(A)** 4th day, **(B)** 8th day, **(C)** 11th day; **(D)** 18th day] after initial symptom. **(A)** Plain CT (computer tomography) brain imaging was normal at the 4th day after initial symptoms. **(B)** Plain CT brain imaging showed hypo-density in the bilateral basal ganglia, bilateral occipital lobe, and left limbic lobe at the 8th day after initial symptoms. **(C)** Plain CT brain imaging showed hypo-density in bilateral basal ganglia, bilateral occipital lobe, bilateral limbic lobe, and left thalamic at the 11th day of initial symptoms. **(D)** Plain CT brain imaging showed hypo-density in bilateral basal ganglia, bilateral occipital lobe, bilateral limbic lobe, and left thalamic at 18th day of initial symptom.

The patient, a 43-year-old man with no prior medical history, who worked as a veterinarian, was admitted to the critical care department of Qilu hospital of Shandong University (Jinan, China) due to high-grade fever of 11 days' duration, headache of 9 days' duration and tonic-clonic seizures as well as coma of 8 days' duration. His hands were punctured by a knife used during the autopsy process of dead swine 4 days before his initial symptom (fever) occurred. At the 3rd day after the initial symptoms, he developed status epilepticus and coma, requiring endotracheal intubation, treatment with intravenous midazolam as well as valproate, and he was treated in the intensive care unit (ICU) of the local hospital for 8 days. Lumbar puncture indicated an opening pressure of 230 mmH_2_O (80–180 mmH_2_O). However, the patient could not provide other detailed results from CSF (cerebrospinal fluid) and serum. On the 4th day after initial symptoms, plain CT (computer tomography) brain imaging was normal (Figure 1A). On 8th day after initial symptoms, plain CT brain imaging showed hypo-density in the bilateral basal ganglia, bilateral occipital lobe and left limbic lobe (Figure 1B). With the suspicion of viral encephalitis, he was started on antiviral therapy (Ribavirin), immunoglobulin and corticosteroids treatment (Methylprednisolone) along with antibiotic therapy (Meropenem and Linezolid) on an empirical basis in the local hospital. He was transferred to our hospital for concerns of infectious etiologies and was immediately empirically started on meropenem, linezolid and acyclovir (10 mg/kg/8 h). On examination during his admission to our hospital, his Glasgow Coma Scale (GCS) was 3/15 (Eye-opening, 1/4; Motor response, 1/6; Verbal response, 1/5). Although he had neck stiffness and Kernig signs, long tract signs, such as hyperreflexia, Hoffman's signs as well as Babinski signs, were not detected. Examination of his cardiovascular, respiratory, and abdominal systems was normal. His vital signs included blood pressure (BP) of 120/70 mmHg, heart rate 100 bpm, temperature 38°C, and SpO_2_ 99% on inspired oxygen at 35% with mechanical ventilation. In addition, spontaneous breaths were not detected. All antibiotics were discontinued at the 3rd day of treatment at our hospital and only antiviral therapy [acyclovir (10 mg/kg/8 h)] was preserved, for a total of 2 weeks. After 1 month of treatment, he was still dependent on tracheostomy and gastrostomy tubes, but has been weaned off mechanical ventilation. The patient was voluntarily discharged to another hospital for further rehabilitation of neurological function.

## Lab Findings

On examination during his admission to our hospital, his laboratory findings were hemoglobin (Hb) 9.3 g/dL (115–150 g/dl), white cell count (WBC) 6.39^*^10^9^/L (3.5–9.5^*^10^9^/L), platelet count 120^*^10^9^/L (125–350^*^10^9^/L), sodium 140 mmol/L (135–145mmol/l), potassium 3.6 mmol/L (3.5–5.5mmol/l), creatinine 89 μmol/L (62–115 μmol/l), albumin 26.3 g/L (40–55g/l), alanine transaminase (ALT) 155 U/L (9–50 U/l), and aspartate transaminase (AST) 182 U/L (15–40 U/l). Japanese encephalitis virus (JEV)-specific IgM antibody in the plasma was negative. Plain CT brain imaging showed hypo-density in the bilateral basal ganglia, bilateral occipital lobe, bilateral limbic lobe, and left thalamic (Figure 1C). After intensive treatments from the local hospital and our hospital, plain CT imaging showed that the range of hypo-density (Figure 1D) was not less than what was shown by scans on the 8th (Figure 1B) and 11th days (Figure 1C) after initial symptoms. This indicated that the present treatment might not be effective.

**Figure 2:**
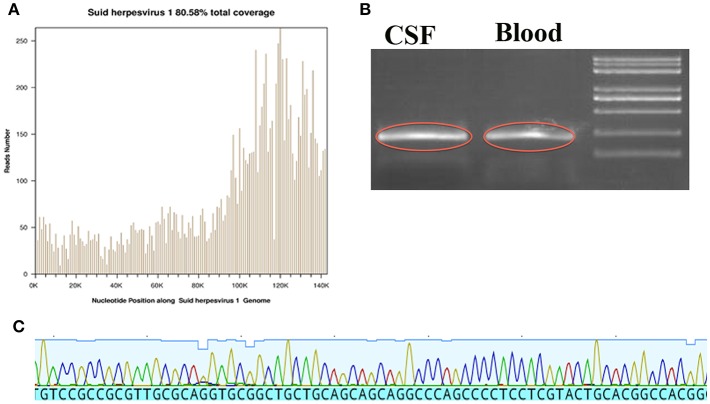
The data from NGS, Sanger sequence and regular PCR. **(A)** The location of detected nucleic acid sequences in PRV, which yielded a total coverage of 80.58%. **(B)** The image outlined in red is the PRV amplified product in CSF and Blood. **(C)** Sanger sequences identified in PRV.

Lumbar puncture revealed transparent fluid with an opening pressure of 90 mmH_2_O (80–180 mmH_2_O), glucose of 1.86 mmol/L (2.8–4.5 mmol/l), protein content of 1.45 g/L (0.15–0.45 g/l), RBC (red blood cell) of 14/ml (0/ml) and WBC (white blood cell) of 189/ml (0–5/ml) (98% monocytes) on the 1st day at our hospital. An infectious disease screen of the central nervous system (CNS) using CSF included PCR against viruses [EBV (Epstein-Barr virus), HSV-1 (Herpes simplex virus type 1), HSV-2 (Herpes simplex virus type 2), CMV (Cytomegalovirus)], Gram and acid-fast stains, cultures and next-generation sequencing (NGS). The CSF screen was negative for current virus and bacterial infection. NGS results showed 6,198 unique sequence reads of PRV in CSF (cerebrospinal fluid), covering 80.58% of the nucleotide sequences (Figure 2A). Sanger sequence analysis in CSF was also performed and confirmed the presence of PRV (Figure 2C). Based on the NGS results, the clinicians suspected that the patient might be infected by PRV. Thus, all antibiotics were discontinued and only antiviral therapy [acyclovir (10 mg/kg/8 h)] was preserved for a total of 2 weeks. In addition, regular PCR (polymerase chain reaction) analysis was also immediately used to further confirm the presence of PRV in CSF as well as blood. As shown in Figure 2B, positive bands were detected in CSF and blood. On the 8th day at our hospital, lumbar puncture showed transparent fluid with an opening pressure of 175 mmH_2_O, glucose of 2.29 mmol/L, protein content of 2.71 g/L, RBC (red blood cell) of 2/ml and WBC (white blood cell) of 174/ml (70% monocytes). NGS was again performed to detect the nucleotide of PRV in CSF. However, no unique sequence reads of PRV were detected in CSF (data not shown).

To further validate our results, we sent the patient plasma and CSF from the 14th day after initial symptom to Dr. Huanchun Chen's lab (the Cooperative Innovation Center for Sustainable Pig Production, Huazhong Agriculture University) for PRV antibody testing. If the antibody titer is not higher than 0.6, the result is considered positive. PRV gB antibody (antibody titer: 0.248) in the serum was positive while PRV gE antibody (antibody titer: 1.001) in the serum was negative. However, Neither PRV gB antibody or PRV gE antibody was detected in the CSF. On the 21st and 28th days after initial symptoms, the patient's plasma was sent again to Dr. Chen's lab for PRV antibody testing. Both PRV gB antibody (separate antibody titers: 0.132 and 0.130) and PRV gE antibody (separate antibody titers: 0.557 and 0.337) in the serum were still positive.

## Discussion

To date, there are no confirmed CNS cases of human PRV infection. There were three suspected CNS cases of human PRV infection after close contact with cats or other domestic animals (3). However, no specific viral nucleic acid in CSF was detected except for serological PRV antibody positive. The first confirmed case of human PRV infection was reported in 2017. The initial symptoms included fever, headache, and visual impairment after handling animal containments. However, no data indicated the presence of CNS infection. To our limited knowledge, we reported for the first time that PRV had the capacity of resulting in encephalitis occurring in man.

The patient's clinical presentation included fever, headache, seizure, and the alternation of consciousness. The characteristics of the patient's CSF included RBC (red blood cell) count of 14, and WBC (white blood cell) count of 189/ml (98% monocytes). The clinical presentation of viral encephalitis is non-specific, including fever, varying degrees of alteration in sensorium with or without focal neurological deficits, and/or seizures. The characteristics of CSF in Herpes simplex encephalitis (HSE) include moderate pleocytosis (usually 10–200 cells/mm^3^), typically mononuclear white and red blood cells, reflecting the hemorrhagic nature of the infectious process within brain parenchyma (4). The patient's clinical presentation and characteristics of CSF were consistent with Japanese encephalitis (JE) or HSE. Thus, at the beginning, the patient in the local hospital was misdiagnosed as having JE or HSE. However, neuroimaging provided arguments for both conditions. HSE mainly affects the limbic cortex, including the basi-frontal and medial temporal cortex while the thalamic, basal ganglia or brainstem are not involved. JE predominantly involves symmetric thalami and substantia nigra (5, 6). The patient's plain CT (Figures 1C,D) showed that the bilateral basal ganglia, right thalamic, and bilateral limbic cortex were involved, which was inconsistent with JE or HSE. Thus, the CT results did not support JE or HSE.

Specific viral diagnosis could be achieved by demonstration of viral nucleic acid or antibody in CSF or isolation of the virus from CSF or brain tissue (7). The sensitivity of traditional CSF tests of CNS infectious diseases is low because the isolation of virus is difficult to accomplish due to the short period of viremia and difficulty in obtaining brain tissue through biopsy. In addition, traditional CSF tests require prior knowledge of possible pathogen identification while NGS offers the possibility of pathogen identification without prior knowledge of the target. Next-generation sequencing (NGS) has been well-accepted as a rapid and precise method for medical microbiology, especially for rare and newly identified CNS viral infection (8). NGS (Figure 2A) and Sanger sequencing (Figure 2C) of the patient found the presence of PRV in CSF, which was a newly identified viral infection in humans. In addition, the data of NGS also showed that the sequences and coverage rate of PRV were high (Figure 2A). Regular PCR results in CSF and blood (Figure 2B) were consistent with NGS results, which further validated the credibility of NGS technology.

Vaccination is thought to be the most important method to control PRV. However, PRV vaccine is still provided for swineherds on a voluntary basis rather than as a requirement in China (2). PRV gE is an envelope glycoprotein that is not essential for virus replication. Additionally, PRV marker vaccines that lack the gene encoding glycoprotein E (gE) are used widely to control or eradiate PRV in piglets in China (9). Thus, the serological tests to detect antibodies specific to gE are able to differentiate infection from vaccinated animals or unvaccinated animals (9). The patient had history of intensive and long-duration contact with swine. The serological results showed that both gB and gE antibodies were positive in plasma, not in CSF, which indicated that not only vaccinated swine but also non-vaccinated swine took part in the viral transmission. Serological (immunological) tests and PCR technology are widely accepted as being able to be used for virological diagnosis (7). Thus, these above results further suggested that the patient had viral encephalitis caused by PRV.

In conclusion, this case of PRV-caused encephalitis indicated that PRV could invade the CNS and infect humans. Clinicians should be especially aware that PRV-caused human encephalitis should be considered in patients with encephalitis of unknown etiology, especially those who have had a history of exposure to swine or other domestic animals. In addition, the broken skin or mucous membrane might make it easy for PRV to transmit from infected animals to human. Not only vaccinated swine but also non-vaccinated swine have the ability of transmitting PRV from pigs to human. Thus, people who worked on animal husbandry and veterinary specialty should increase awareness of self-protection when contacting diseased animals. More importantly, the outcome of PRV-caused encephalitis is poor. And there is no effective drugs to prevent the progress of the disease.

## Patient Consent

We received written informed consent from the patient's wife.

## Author Contributions

HY wrote the manuscript. HH, HW, and YC helped to collect the clinical data and neuroimages. HL and SD helped to send CSF and plama to Dr. Huanchun Chen's lab.

### Conflict of Interest Statement

The authors declare that the research was conducted in the absence of any commercial or financial relationships that could be construed as a potential conflict of interest.
